# Supplementary material for: What Is the Current Direction of the Research on Carotenoids and Human Health? An Overview of Registered Clinical Trials
Source: Nutrients. 2022 Mar 11;14(6):1191. doi: 10.3390/nu14061191 (PMC8955529; doi:10.3390/nu14061191)
Supplement: Supplementary file 1 [file nutrients-14-01191-s001.zip › nutrients-1609674-supplementary-done.pdf]

nutrients MDPI

Supplementary Table S1: "List of the registered clinical trials."

Rank	Trial Number	Title
1	ISRCTN18206561	The efficacy of different nutritional formulas of lutein, zeaxanthin, and meso-zeaxanthin and their impact on the human body
2	ISRCTN13244115	Effect of a tomato sauce (OsteoCol) from vine-ripened tomatoes on lipids
3	ISRCTN16156382	Effect of Lutemax 2020 on blue light and visual health markers
4	ISRCTN17604865	Effect of Lutemax 2020 on visual and brain health markers
5	ISRCTN15646478	A randomized, double-blind, placebo-controlled study to investigate the effects of OmniActive Lutemax 2020® oil suspension on skin health attributesA randomized, double-blind, placebo-controlled study to investigate the effects of OmniActive Lutemax 2020
6	ISRCTN54990825	Effects of three levels of macular carotenoid supplementation on macular pigment optical density, psychological stress levels, and overall health
7	ISRCTN86297454	Can an increase in fruit and vegetable intake improve/change your skin colour and appearance?
8	ISRCTN56985060	Macular Pigment and Glaucoma Trial
9	ISRCTN25867083	Egg Xanthophyll Interventional Trial
10	ISRCTN99660610	Assessment of different doses of tomato intake on blood pressure and endothelial function in patients at high vascular risk: nutrigenomics effect on different phenotypes of risk
11	ISRCTN68270512	Central Retinal Enrichment Supplementation Trials - enrichment of macular pigment with respect to vision in Normal subjects
12	ISRCTN42483402	Amyloid β levels in human red blood cells
13	ISRCTN55089134	The effect of the dietary antioxidant supplements lutein and zeaxanthin on retinal macular pigment optical density
14	ISRCTN10624844	Lutein and zeaxanthin supplementation in preterm infants to prevent retinopathy of prematurity
15	ISRCTN34203810	Effects of tomato-based foods on cardiovascular disease risk
16	ISRCTN29971311	Lutein supplementation in very low birth weight (VLBW) neonates in neonatal intensive care units (NICU)
17	ISRCTN81595685	Age-related macular degeneration Meso-zeaxanthin Ocular Supplementation trial (AMOST)
18	ISRCTN60816411	Meso-zeaxanthin Ocular Supplementation Trial (MOST)
19	NCT00006202	Lutein for Age-Related Macular Degeneration
20	NCT00006078	Lycopene In Preventing of Prostate Cancer
21	NCT04744233	Bioavailability of Carotenoids From Orange Juice in a Cross-over Study in Healthy Subjects.
22	NCT00198822	Impact of Maternal Vitamin A or Beta-Carotene Supplementation on Maternal and Infant Mortality in Bangladesh
23	NCT00029289	Effects of Lutein in Retinitis Pigmentosa

24	NCT00156169	The Effect of the Alga Dunaliella Bardawil as a Source of 9-cis Retinoic Acid on Lipid Profile in Fibrate Treated Patients.
25	NCT00356252	Biological Functions of Carotenoids in Humans
26	NCT00275418	Beta Carotene From Natural Source for Patients With Non-Active Crohn's Disease
27	NCT02292524	Prostate Biomarkers in Men Consuming Tomato Products
28	NCT00128752	The CARO Study - Bioefficacy of Beta-Carotene in Oil and in a Mixed Diet in Healthy Subjects
29	NCT00346333	Clinical Trial of Lutein for Patients With Retinitis Pigmentosa Receiving Vitamin A
30	NCT00178113	A Pilot Study of Lycopene Supplementation in Prostatic Intraepithelial Neoplasia
31	NCT00402285	Lycopene or Omega-3 Fatty Acid Nutritional Supplements in Treating Patients With Stage I or Stage II Prostate Cancer
32	NCT00680277	Vitamin A Value of Spirulina Carotenoids in Humans
33	NCT00680355	Bioavailability of Golden Rice Carotenoids in Humans
34	NCT00718653	Effects of Antioxidants on Human Macular Pigments
35	NCT00128804	The STOVITA Study - Bioefficacy of Beta-Carotene in Oil and in a Mixed Diet in Ileostomy Patients
36	NCT00154843	A Clinical Study to Determine Factors Affecting Absorption and Serum Levels of Lycopene After Supplementation
37	NCT00068731	Lycopene in Treating Patients With Metastatic Prostate Cancer
38	NCT00093561	Lycopene in Preventing Prostate Cancer in Healthy Participants
39	NCT00656214	Treatmant of Oral Lichen Planus With Lycopene
40	NCT00672230	Dietary Lutein and the Retinopathy of Prematurity
41	NCT00913406	Effect of Lutein in S-26 Gold On Growth And Safety
42	NCT00879671	Effects of Lutein Supplementation on Macular Pigment Optical Density and Visual Acuity in Patients With Age-related Macular Degeneration
43	NCT01443026	The Effects of Lycopene on High Risk Prostatic Tissue
44	NCT00450957	Lycopene in Healthy Male Participants
45	NCT00322114	Lycopene in Preventing Prostate Cancer in Healthy Participants
46	NCT00172367	Chemoprevention Trial for Uremia-Associated Urothelial Carcinoma
47	NCT00449917	Visiobiane Anti-age Effects on Vision Parameters
48	NCT00453648	Impact of Consumption of Orange-fleshed Sweet Potatoes on the Vitamin A Status of Bangladeshi Women of Reproductive Age
49	NCT02348164	Human Lycopene and Beta-cryptoxanthin Absorption From Citrus Fruit
50	NCT00596024	Lutein and Alzheimer's Disease Study
51	NCT01042860	The Influence of Lutein Supplements on Age-related Macular Degeneration
52	NCT00564902	The Zeaxanthin and Visual Function Study
53	NCT00636350	Bioavailability of Pepper Carotenoids in Humans
54	NCT00569023	Treatment of Congenital Stationary Night Blindness With an Alga Containing High Dose of Beta Carotene
55	NCT00527553	The Effect of Eggs and Egg Products on Macular Pigment

56	NCT00539916	Effects of Regular and Consequent Citrus Fruits Consumption on Vascular Protection
57	NCT00680212	Vitamin A Equivalence of Plant Carotenoids in Children
58	NCT00636038	Bioavailability of Yellow Maize Carotenoids in Humans
59	NCT00450749	Lycopene in Treating Patients Undergoing Radical Prostatectomy for Prostate Cancer
60	NCT01256697	The Effect of Oral Administration of 9-cis Rich Powder of the Alga Dunaliella Bardawil on Visual Functions im Patients With Retinitis Pigmentosa
61	NCT00937742	The Effect of Processed Tomato Products on CVD Risks
62	NCT01048476	Effects of Lutein and Zeaxanthin Supplementation on Age-related Macular Degeneration
63	NCT00872664	Skin and Serum Carotenoids in Preterm Infants Fed on a Formula Supplemented With Carotenoids
64	NCT00909090	Macular Pigment and Glare Disability
65	NCT00902408	Effect of Lutein-enriched-egg Beverage on Age-Related Macular Degeneration (AMD)
66	NCT01175577	Feeding the Rainbow to Investigate Endothelial Dysfunction
67	NCT01221558	Effects of Lycopene on Oxidative Stress and Markers of Endothelial Function Healthy Men
68	NCT01105338	Lycopene or Green Tea for Men at Risk of Prostate Cancer
69	NCT00939237	Trial of Lycopene/Ateronon for Secondary Prevention of Coronary Heart Disease
70	NCT01142960	Effects of Lycium Barbarum Supplements on the Progression of Senile Cataract in Hong Kong Chinese Population
71	NCT00948363	The Effect of Kiwi on Blood Pressure, Endothelial Function, Antioxidant Capacity and Gene Expression
72	NCT01342666	Tomato Consumption and High Density Lipoprotein-cholesterol
73	NCT01528605	Effects of Lutein and Zeaxanthin Supplementation on Early Age-related Macular Degeneration
74	NCT01534533	Effects of Lutein Supplementation on Subclinical Atherosclerosis
75	NCT03945526	Effect of Astaxanthin Supplementation on Plasma Malondialdehyde Levels and NIHSS of Stroke Patients
76	NCT01316198	Effects of Xanthophylls on Optical Density
77	NCT01056094	Effects of Lutein Supplementation on Oxidative Stress and Inflammation in Healthy Nonsmokers
78	NCT01420406	Vitamin A Bioavailability in Lactating Women With Marginal Vitamin A Status
79	NCT01477112	Low Dose β-carotene Supplementation Diminishes Oxidative Stress in Type 2 Diabetics and Healthy Individuals
80	NCT00905931	Lycopene Following Aneurysmal Subarachnoid Haemorrhage
81	NCT01241877	Astaxanthin Supplementation in Cyclists
82	NCT01167205	Positive Effects of Haematococcus Astaxanthin on Oxidative Stress and Lipid Profile in Overweight and Obese Adults

83	NCT01121081	The Effect of Algae Dunaliella Bardawil on Psoriasis (2)
84	NCT01100385	TomVasc - Vascular Effects of Tomato Extract
85	NCT02068807	Evaluation of Antioxidant Activity of Oral Lutein in Preterm and Term Newborn
86	NCT01748916	Comparison of Carotenoid Bioavailability From Fresh Papaya, Tomato and Carrot
87	NCT01403844	Carotenoid Response to the Intake of Vegetables and Fruits
88	NCT01400763	Lutein Bioavailability From Fresh and Dried Beverages
89	NCT01381276	Vitamin A Absorption From Cassava in Women
90	NCT01432210	Provitamin A Absorption and Conversion With Avocados
91	NCT01323036	Krill Oil Study Compared to Fish Oil
92	NCT01462825	The Effect of Tomato Ketchup on Inflammation
93	NCT02276014	Effect of SNPs in the BCMO1 Enzyme
94	NCT02023645	The Effects of a Carotenoid Intervention on Cognitive Function
95	NCT01692340	Novel 13C Carotenoids for Absorption and Metabolism Studies in Humans
96	NCT01730898	Lutein Absorption in Healthy Adults
97	NCT01696773	Carotenoid and Flavonoid Absorption From Red and Tangerine-Type Tomatoes
98	NCT01695148	Efficacy of β-carotene Biofortified Maize in Reducing Vitamin A Deficiency Among Children
99	NCT01665469	Effect of Tomato Extracted Lycopene on Postprandial Oxidation and inflammation in Healthy Weight Men and Women
100	NCT01680510	The Effect of Oral Administration of 9-cis β Carotene Rich Powder of the Alga Dunaliella Bardawil
101	NCT01667510	Effect of Cardio Mato (Grade A Lyc-O-Mato, a Tomato Extracted Lycopene) on Blood Pressure
102	NCT01726127	Green Vegetables and Women's Health
103	NCT01621646	The Effect of Daily Consumption of Eggs on Cognitive Function in the Elderly
104	NCT01620567	Consumption of Potatoes, Avocados and Chickpeas and Cognitive Function in Older Adults
105	NCT01628081	The Effect of Alga Dunaliella Bardawil on Psoriasis
106	NCT01614483	Efficacy of Yellow Cassava to Improve Vitamin A Status of Kenyan School Children
107	NCT01803659	Effect of Small Daily Doses of B-carotene on Breast Milk Retinol
108	NCT02257801	Effects of Lutein on Learning and Memory in Chronically Stressed Subjects and in an Acute Stressful Situation (TSST - Trier Social Stress Test): A Randomized, Double-blind, Placebo-controlled Trial)
109	NCT01922713	Effect of Daily Consumption of Orange Maize on Breast Milk Retinol in Lactating Zambian Women
110	NCT02587117	A Clinical Trial to Study the Effects of Two Drugs, Lycopene and Prednisolone in Patients With Oral Lichen Planus
111	NCT02263352	A Clinical Trial to Study the Effect of Lycopene in Patients With Gum Disease and type2 Diabetes Mellitus
112	NCT02263183	Red Palm Olein and Cardiovascular Health

113	NCT01968369	Effects of Tomato Sauce on Endothelial Dysfunction Induced by a High Fat Meal in Healthy Subjects
114	NCT03492593	Lycopene and Beta-carotene Metabolism in the Digestive Tract of Healthy Men
115	NCT02200263	The Effects of Lutein and Zeaxanthin Supplementation on Vision in Patients With Albinism
116	NCT02246985	Bioavailability of Carotenoids Added Into Processed Foods
117	NCT01951313	Effects of Egg Consumption on Carotenoid Absorption From Co-consumed, Non-Egg Food
118	NCT02210507	Beta-carotene Absorption and Bioconversion to Vitamin A From Biofortified Cassava Gari
119	NCT02069522	Healthy Term Infants Fed Milk-Based Formulas
120	NCT02088242	Physiological and Molecular Influences of Astaxanthin Supplementation on Heat Strain in Humans
121	NCT02380144	Bioavailability of Carotenoids and Flavonoids From Fresh Oranges and Orange Juice.
122	NCT02113254	Study of the Macular Pigment by the Consumption of Corn With Strong Content in Zeaxanthine
123	NCT02343497	Lipid-lowering Effects of an Astaxanthin Supplement in Volunteers With Mild Dyslipidaemia
124	NCT02369666	LycoRed 40051 Product as a Countermeasure to Exercise Stress
125	NCT04015544	Markers of Atherosclerosis in Overweight, Postmenopausal Women Following Daily Watermelon Consumption
126	NCT02468882	Does Watercress Intake Have an Impact on Cancer Patients Outcomes: a Longitudinal Trial
127	NCT03932305	Randomized Controlled Trial of LUtein as a Novel Neuroprotective Adjunctive Therapy to Improve Visual Outcome of Rhegmatogenous Retinal Detachment (LUNAR Study)
128	NCT02550483	Novel Metabolites of Beta-Carotene and Lycopene
129	NCT02450227	Carotenoid Absorption and Metabolism From Green-leafy Vegetable Matrices
130	NCT02136303	Regional Kale as Source of Lutein and Zeaxanthin to Improve Vision of AMD-patients
131	NCT02373943	The MULTINUTRIENT Maize Project: Results of Human Feeding Trial
132	NCT02679794	Impact of Egg Consumption on Carotenoid and Vitamin D Bioavailability in Pre- and Post-menopausal Women
133	NCT02379169	Effects of Sea Buckthorn Oil and Lutein on Eye Health
134	NCT02397811	Astaxanthin Formulation Bioavailability
135	NCT03203746	Gingival Crevicular Fluid Levels of Protein Carbonyl Following the Use of Lycopene in Chronic Periodontitis
136	NCT01102504	Supplementation of Lycopene in Carotid Atheroma
137	NCT02144649	Tangerine or Red Tomato Juice in Treating Patients With Prostate Cancer Undergoing Surgery

138	NCT04306406	Molecular Mechanisms of Raspberries Effect on Insulin Resistance and Inflammation
139	NCT03820245	Effect of Short-term Annatto Carotenoids Supplementation on Oxidative Stress Status in Healthy Individuals
140	NCT02800408	Comparison of Beta-cryptoxanthin Bioavailability From Biofortified Maize in Humans
141	NCT03310359	Clinical Trial of Anti-oxidant Astaxanthin in Insulin-resistant Subjects
142	NCT03167268	Panitumumab Skin Toxicity Prevention Trial
143	NCT03463967	Effects of Tomato Products in Children With NAFLD
144	NCT02804490	Efficacy of Biofortified Maize to Improve Maternal and Infant Vitamin A Status
145	NCT03520725	Antioxidant Capacity and Vitamin A Profile in Young Adults Who Consume a Snack Bar Made From Tropical Fruits Treated With UV-C
146	NCT03581955	Identification of New Biomarkers of Banana and Tomato Intake
147	NCT02949115	Effects of Red Beetroot Juice on High-Fat Meal-Induced Endothelial Dysfunction and Cardiometabolic Disturbances
148	NCT02740439	Persea Americana for Total Health (PATH) Study
149	NCT03626168	Bioactive Compounds in Watermelon Modulating Oxidative Stress and Inflammation in Elders
150	NCT03113864	Effects of Lutein on Visual Function
151	NCT03873909	Bioavailability of Carotenoids Present in Mamey Sapote (Pouteria Sapota (Jacq.) H. E. Moore & Stearn) Fruit
152	NCT04460365	European Nutrition in Glaucoma Management Trial
153	NCT03237702	Decipher Lethal Prostate Cancer Biology - Urine Metabolomics
154	NCT03209817	Tomato Supplementation and Cardiovascular Health
155	NCT03256123	The Effects of Biscuits Containing Red Palm Oil on School Children With Vitamin A Deficiency in West and East Malaysia
156	NCT02903433	Effects of Avocado Intake on the Nutritional Status of Families
157	NCT03340103	Early Administration of the Lutein/Zeaxanthin in Premature Newborns
158	NCT03807050	Safety and Pharmacokinetics of Phaffia Rhodozyma Astaxanthin
159	NCT04741763	Night Vision and Carotenoids
160	NCT03202043	The Juice Study: Sensitivity of Skin Carotenoid Status to Detect Change in Intake
161	NCT04652739	Evaluation of Lycopene in the Treatment of Erosive Oral Lichen Planus
162	NCT03521349	Correlational and Intervention Effects of Egg Consumption on Macular Carotenoids, Cognition, and Achievement During Childhood
163	NCT03964935	Lycopene vs Minocycline Hydrochloride as Adjunctive to Periodontal Treatment
164	NCT03443882	Bioavailability of Astaxanthin Formulations
165	NCT03590756	Influence of Mango Intake of Skin Health in Postmenopausal Women
166	NCT04583722	Evaluating Astaxanthin Bioavailability, and a New Technology for Improving it, Using Natural Food Materials Only
167	NCT03535844	Cardio-vascular Protective Effects of Wolfberry in Middle-aged and Older Adults
168	NCT04153968	Bioefficacy of Beta-cryptoxanthin From Biofortified Maize

169	NCT03959592	Macular Pigment and Visual Performance in Glaucoma Patients
170	NCT03811977	The Effect of 12-Week Dietary Intake of Lutein on Minimal Erythema Dose and Other Skin Parameters
171	NCT04078646	Influence of Proteins on the Bioavailability of Carotenoids
172	NCT03983525	The Effect of Gojiberry Intake (Lycium Barbarium) on Macular Function
173	NCT03769779	Evaluation of the Benefits of FloraGLO™ Lutein on Skin Health
174	NCT03783013	Tomato Juice for Health Study
175	NCT03773497	Bioavailability of Phytonutrients From Novel Preparations of Broccoli
176	NCT03838536	Synergistic Activity of Human Milk Nutrients and Infant Cognition
177	NCT03951883	Does Increased Egg Consumption Have Cognitive and Neural Benefits in Food Insecure, At-risk Adolescents?
178	NCT03825276	Effect of Mango on Gut Microbiota and Metabolic Health
179	NCT03991286	The Effect of Astaxanthin on Hormonal Parameters and Oxidative Stress Indices in Patients With Polycystic Ovary Syndrome
180	NCT04689984	The Benefits of Astaxanthin as Add on Therapy in the Management of Painful Diabetic Neuropathy Patient
181	NCT04234334	Benefits of Eggs in Combination With a Plant-based Diet
182	NCT04786392	Food or Supplemental Lutein Absorption
183	NCT04843813	Effects of Lutein Supplementation on Cognition and MPOD in Multiple Sclerosis Patients-
184	NCT04056624	Examining Validity and Sensitivity of Pressure-Mediated Reflection Spectroscopy
185	NCT03664466	Astaxanthin Effects on Osteoarthritis Associated Pain and Inflammatory Indicators
186	NCT02018692	The Effect of Oral Administration of 9-cis Rich Powder of the Alga Dunaliella Bardawil on Visual Functions in Adolescent Patients With Retinitis Pigmentosa
187	NCT04869852	Effects of Mango Intake on Skin Health and Gut Microbiome Changes in Postmenopausal Women
188	NCT04792216	Salmon Intake and Gut Health in Adults
189	NCT05046457	Beta-cryptoxanthin Supplement: Absorption and Function
190	NCT05015374	Effect of Astaxanthin on the Patients With Alzheimer Disease
191	NCT05009810	Efficacy and Safety of Astaxanthin in Volunteer With Refraction Errors
192	NCT05064865	Impact of Regular Consumption of Grapes on Eye Health in Singapore Older Adults
193	NCT04974450	Trigona Honey and Intestinal Microbiota Among Obese People
